# Remote Assessment of Functional Mobility and Strength in Older Cancer Survivors: Protocol for a Validity and Reliability Study

**DOI:** 10.2196/20834

**Published:** 2020-09-01

**Authors:** Cindy K Blair, Elizabeth Harding, Carla Herman, Tawny Boyce, Wendy Demark-Wahnefried, Sally Davis, Anita Y Kinney, Vernon S Pankratz

**Affiliations:** 1 Department of Internal Medicine University of New Mexico Albuquerque, NM United States; 2 Comprehensive Cancer Center University of New Mexico Albuquerque, NM United States; 3 Department of Nutrition Sciences University of Alabama at Birmingham Birmingham, AL United States; 4 O’Neal Comprehensive Cancer Center University of Alabama at Birmingham Birmingham, AL United States; 5 Department of Pediatrics University of New Mexico Albuquerque, NM United States; 6 Prevention Research Center University of New Mexico Albuquerque, NM United States; 7 Department of Biostatistics and Epidemiology School of Public Health Rutgers University Piscataway, NJ United States; 8 Rutgers Cancer Institute of New Jersey Rutgers University New Brunswick, NJ United States

**Keywords:** physical function, physical performance, older adults, remote assessment, videoconferencing, cancer survivors, cancer, elderly, physical activity, telehealth

## Abstract

**Background:**

Older cancer survivors, faced with both age- and treatment-related morbidity, are at increased and premature risk for physical function limitations. Physical performance is an important predictor of disability, quality of life, and premature mortality, and thus is considered an important target of interventions designed to prevent, delay, or attenuate the physical functional decline. Currently, low-cost, valid, and reliable methods to remotely assess physical performance tests that are self-administered by older adults in the home-setting do not exist, thus limiting the reach, scalability, and dissemination of interventions.

**Objective:**

This paper will describe the rationale and design for a study to evaluate the accuracy, reliability, safety, and acceptability of videoconferencing and self-administered tests of functional mobility and strength by older cancer survivors in their own homes.

**Methods:**

To enable remote assessment, participants receive a toolkit and instructions for setting up their test course and communicating with the investigator. Two standard gerontologic performance tests are being evaluated: the Timed Up and Go test and the 30-second chair stand test. Phase 1 of the study evaluates proof-of-concept that older cancer survivors (age ≥60 years) can follow the testing protocol and use a tablet PC to communicate with the study investigator. Phase 2 evaluates the criterion validity of videoconference compared to direct observation of the two physical performance tests. Phase 3 evaluates reliability by enrolling 5-10 participants who agree to repeat the remote assessment (without direct observation). Phase 4 enrolls 5-10 new study participants to complete the remote assessment test protocol. Feedback from participants in each phase is used to refine the test protocol and instructions.

**Results:**

Enrollment began in December 2019. Ten participants completed the Phase 1 proof-of-concept. The study was paused in mid-March 2020 due to the COVID-19 pandemic. The study is expected to be completed by the end of 2020.

**Conclusions:**

This validity and reliability study will provide important information on the acceptability and safety of using videoconferencing to remotely assess two tests of functional mobility and strength, self-administered by older adults in their homes. Videoconferencing has the potential to expand the reach, scalability, and dissemination of interventions to older cancer survivors, and potentially other older adults, especially in rural areas.

**Trial Registration:**

ClinicalTrials.gov NCT04339959; https://clinicaltrials.gov/ct2/show/NCT04339959

**International Registered Report Identifier (IRRID):**

DERR1-10.2196/20834

## Introduction

Over 16.9 million cancer survivors are living in the United States, and three-quarters are 60 years of age or older [1]. Compared with individuals without a history of cancer, cancer survivors are at increased and premature risk of developing age-related diseases and conditions [2]. It is hypothesized that cancer and its treatment can lead to “a paralleled ‘normal’ aging trajectory with weakened physiologic reserve (Phase Shift or Accentuated Aging Hypothesis) or an altered aging trajectory with quicker progression to functional decline (Accelerated Aging Hypothesis)” [3]. Older cancer survivors, faced with both age- and treatment-related morbidity, are at increased and premature risk for physical function limitations [4-7]. Compared with the general population, cancer survivors have a two- to five-fold increased risk of having one or more functional limitations [6]. The adverse consequences of functional limitations, especially mobility limitations, include an increased number of falls, hospital/nursing home admissions, diminished quality of life, premature death, and substantial financial costs [8-11]. Thus, interventions have been designed to improve physical functioning or at least attenuate functional decline among older cancer survivors. However, the majority of interventions that collect objective measures of physical functioning require the participant to travel to the research center or clinic for the assessment even when interventions are provided at home. This requirement can result in selection and attrition bias and limits the reach, scalability, and dissemination of interventions.

Unlike self-reported physical function, the collection of objective measures of physical function (ie, physical performance) requires more resources. It has been challenging to collect objective measures of physical function outside of a standardized environment. Common objective measures of physical performance in older adults include the Short Physical Performance Battery, and the Rikli and Jones Senior Fitness Test that assess mobility, strength, balance, agility, endurance, and postural control [12-14]). Traditionally, these objective measures are collected in a clinic or research setting, to standardize both basic test equipment, such as an armless chair of specified height for chair stand tests, and walking courses, which require adequate space that is free of interruptions and fall hazards. Additionally, specialized equipment is more readily available, such as a hand dynamometer for measuring grip strength or motion capture systems for measuring gait and balance. Another advantage of evaluating physical performance in the clinic is the opportunity for trained staff to provide spotting during more advanced tests or for individuals at greater risk of falls or other injuries. However, a major disadvantage of requiring clinic visits is the travel burden experienced by study participants, which may exclude older, rural, or other individuals unable or unwilling to travel for a research study.

One alternative to assessing physical functioning in a standardized setting is to use study staff or community health workers to conduct home visits to collect objective data. However, this can be time-consuming, costly, and difficult to staff, especially if studies include rural-dwelling participants from large geographical areas. Another option is to utilize technology to remotely assess physical performance, that is, collection of physical performance data that does not require travel for either the participant or study staff. For this option to be practical, the technology would need to produce valid and reliable results. Additionally, the technology should be inexpensive, easy for study participants to use, and straightforward for researchers to score and interpret the test results. Moreover, it is critical that such testing be safe.

The past decade has seen tremendous advances in the development and testing of wearable sensors to evaluate important outcomes like physical performance. However, to date, wearable sensors are either very expensive [15-18], proprietary [19,20], require technicians present for testing [15,17,21], and/or involve complex programming code to process the data [22]. For example, commercially available systems that include wearable sensors, such as LEGSys or the Opal, and software to administer and score standard gait tests (eg, Timed Up and Go, or TUG test), provide a range of useful gait parameters [23,24]. However, the cost of each sensor plus the cost to administer and score each performance test is not economical for most physical activity intervention trials.

Smartphone apps with instrumented versions of physical performance tests have been developed and show great promise [22,25-29]. In particular, instrumented versions of the Timed Up and Go test (iTUG) and the 30-second chair stand test (30s-CST), have been developed for either android phones or iPhones. An advantage to this technology is the ability to capture the sub-phases of a test, thus providing information regarding the quality of movement, in addition to providing more accurate quantification of the movement tasks (ie, time in seconds to complete the test or the total number of stands). For example, some apps can distinguish between the sit-to-stand and stand-to-sit subphases of the chair stand test [30], or the sit-to-stand, walk, turn, walk, stand-to-sit subphases of the iTUG test [28-30]. To date, most of these instrumented performance measures are still being evaluated in large studies, such as the PreventIT trial in Europe [30], have been commercialized (and thus are more expensive) [28,29], or are still being refined and evaluated [26,27,31,32].

Furthermore, to our knowledge, only one study has evaluated the self-administration of the instrumented tests by study participants in the home setting [26]. As noted by Bergquist and colleagues, different usability problems arise when moving the evaluation of an instrumented performance test from the lab to a home-setting [26]. If the apps are intended for unsupervised use in the home setting, then the validity, reliability, usability, and acceptability should be evaluated in that same setting. Thus, further studies are needed before these apps are ready and available for widespread use.

Before COVID-19, telerehabilitation studies have been conducted using videoconferencing to assess physical performance; however, these studies have included a technician or study investigator in the same room or (hospital) building as the study participant during the tests [33-36]. Additionally, few if any of these studies have been tested in rural areas, which often have less reliable high-speed internet necessary for quality video transmission. Similarly, home-based rehabilitation interventions delivered via technology typically have involved an in-person assessment of physical performance, either through home visits by a member of the study team [37,38] or by requiring participants to travel to the research center for data collection [39,40].

At the time our study was designed (pre–COVID-19), low-cost, valid, and reliable methods to remotely assess physical performance tests that are self-administered by older adults in the home setting were not readily available to researchers. Therefore, we propose to use an existing, low-cost, and easy to use technology (videoconferencing) to remotely assess tests of functional mobility and strength that are self-administered by older (≥60 years) cancer survivors. The primary objective of this study is to evaluate the validity, reliability, acceptability, and, most importantly, the safety of having the participants perform two standard gerontologic physical performance tests in their own homes with remote assessment via videoconferencing. These results will be compared to the traditional direct observation (ie, in-person observation and scoring of tests) and accelerometer data. We hypothesize that older cancer survivors, in the presence of a family member or friend, will complete the physical performance self-assessment in the home environment. We further hypothesize that the agreement between the videoconferencing method and the traditional direct observation approach will be within a clinically acceptable limit. The purpose of this paper is to present the research protocol for the validity and reliability study.

## Methods

### Overall Study Design

**Figure 1:**
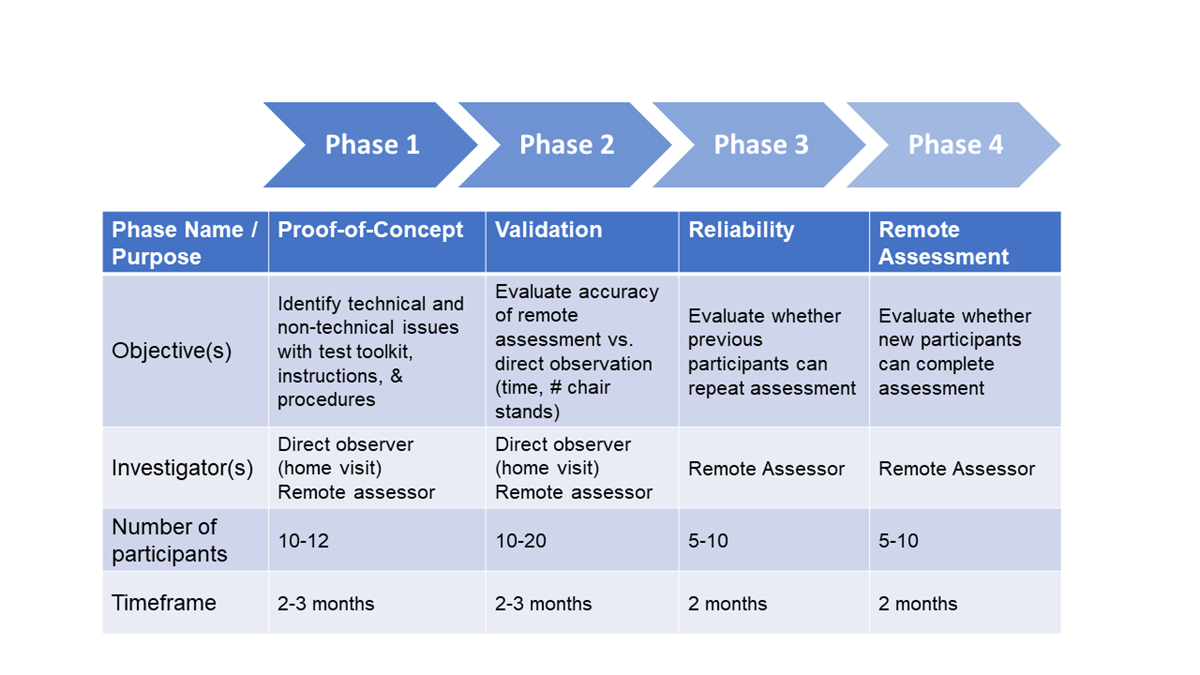
Phases of the remote assessment study.

The ultimate goal of this research study is to develop a test protocol to allow older cancer survivors to self-administer two tests of functional mobility and strength in their own homes. At the same time, an investigator remotely assesses the tests via videoconferencing. This objective is being achieved over a series of phases. Phase 1 (Proof-of-Concept) will evaluate proof-of-concept that older cancer survivors can follow the testing protocol and use a tablet PC to communicate with a remote assessor. Phase 2 (Measurement Validity) will evaluate the validity of videoconference assessment vs direct observation of physical performance tests. Phase 3 (Reliability) will evaluate the reliability by enrolling a sample of participants from earlier phases to repeat the assessment. Participant feedback from each phase will be used to revise the test protocol and instructions. Phase 4 (Remote Assessment) will enroll new study participants who will complete the revised test protocol and undergo de novo remote assessment of physical performance (see Figure 1 for an overview; see procedures below for details). The Human Research Review Committee at the University of New Mexico Health Sciences Center approved the study.

### Setting

All testing takes place inside the study participants’ homes, in a room and or hallway with adequate space to safely perform the two tests of functional mobility and strength. For Phases 1 and 2, one or more investigators are in the home, directly observing and timing the tests for comparison with the remote assessor. For all phases, an investigator serving as the remote assessor is located offsite.

### Participants

Convenience sampling is used to recruit participants for this validity and reliability study. Study flyers are being distributed in areas frequented by older adults, such as primary care clinics, libraries, senior and community centers, and cancer support groups. Additionally, individuals from previously completed studies providing permission for future contact are mailed a letter explaining the new study and inviting them to participate. Individuals expressing interest in the study are assessed for eligibility during a screening telephone call.

Eligibility criteria for the current study are primarily based on criteria to be used in future physical activity interventions aimed at improving physical functioning in older cancer survivors. The criteria include (1) men and women aged 60 years and older, residing in New Mexico; (2) previous diagnosis of cancer (any site, any stage); 3) ≥2 physical function limitations (≥2 functions limited a lot or limited a little on the SF36 Physical Function Subscale, which includes 10-items ranging from self-care to vigorous-intensity activities) [41,42]; (4) able to speak, read, and understand English; (5) participating in less than 120 minutes per week of moderate-to-vigorous physical activity, ie, not meeting physical activity guidelines and accounting for potential over-estimation due to self-report bias. A modified version of the Godin Leisure-Time Exercise Questionnaire that also includes duration is used. Frequency is multiplied by duration for each reported moderate- and strenuous-intensity activity and summed to determine the weekly amount of minutes; (6) living independently and capable of walking three blocks (approximately 1/4 mile or 1300 steps) without stopping to rest; (7) no severe impairments or pre-existing medical limitations for engaging in daily light physical activity (eg, severe orthopedic conditions, dementia, chronic vertigo); (8) no severe hearing, cognitive, or vision deficits that would inhibit communication with the research team via videoconferencing and tablet use; (9) willing to use a tablet computer and videoconferencing software to communicate with a study team member during the assessment; (10) adequate space (minimum of 13 feet by 3 feet) to safely conduct the physical performance tests; (11) availability of a family member or friend to be present (for safety) during remote assessment of performance tests (Phases 3 and 4 only; for Phases 1 and 2, a study team member serves as a safety check when a friend or family member cannot be present during the assessment); and (12) not at high risk for falls (determined using a subset of questions from the Falls Efficacy Scale–International). Individuals who are at high risk for falls and ineligible are asked if they would like more information on Fall Prevention and if yes, they are mailed a brochure from the CDC STEADI Program [43].

Written informed consent for those interested and study eligible is obtained via mail or through REDCap eConsent. Upon receipt of written informed consent, participants are scheduled for the home visit and remote assessment (Phases 1 and 2) or delivery of the test toolkit and subsequent remote assessment (Phases 3 and 4). Prior to the assessment, participants are mailed a location and materials checklist and a 10-foot tape measure. The checklist includes recommendations for choosing a location in their home to safely conduct the tests of functional mobility and strength (Multimedia Appendix 1).

### Protocol for the Remote Assessment of Functional Mobility and Strength

#### Functional Tests

The tests include the TUG test and the 30s-CST. Both of these tests are included in the CDC Stopping Elderly Accidents, Deaths, and Injuries (STEADI) toolkit for assessment of falls [43,44]. These two performance tests incorporate movements typically undertaken during normal daily activities (standing from a chair, walking a short distance, sitting on a chair), and thus represent tasks that are more likely to be safely performed in a clinically unsupervised setting. Both the 30s-CST and TUG tests have been routinely conducted in adult populations with functional limitations such as cerebral palsy, Chronic obstructive pulmonary disease, knee osteoarthritis, low back pain, multiple sclerosis, Parkinson’s disease, renal transplant, rheumatoid arthritis, stroke, vestibular disorders, and frail elderly [45,46].

The TUG test is measured as the time to stand from a standard chair, walk 10 feet, turn around (180° turn), return to the chair, and sit down [44,47]. The TUG test is a measure of mobility and balance and has good validity (0.6<r<0.85) [47,48], and excellent reliability (intraclass coefficient, ICC>0.95) [47,49,50]. This timed test is to be performed as quickly and safely as possible. The 30s-CST involves standing up from a chair and sitting down as quickly and safely as possible, preferably without the use of upper extremity support [44,51]. It is measured by the number of times a person comes to a full standing position from a chair in 30 seconds. The 30s-CST is a measure of lower extremity strength and dynamic balance and has good validity (0.7<r<0.8) and excellent reliability (ICC 0.84-0.92) [51]. Both tests are timed with a stopwatch.

#### Test Tool Kit

**Figure 2:**
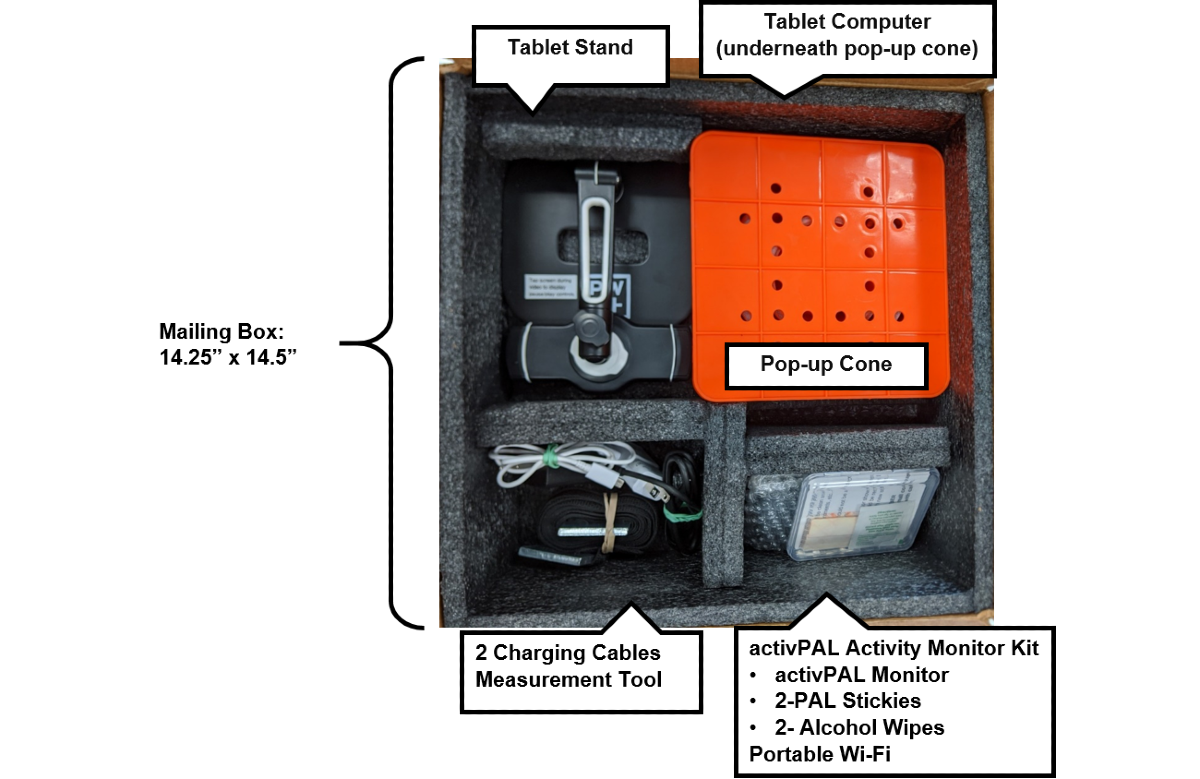
Toolkit for the remote assessment of functional mobility and strength among older cancer survivors.

**Figure 3:**
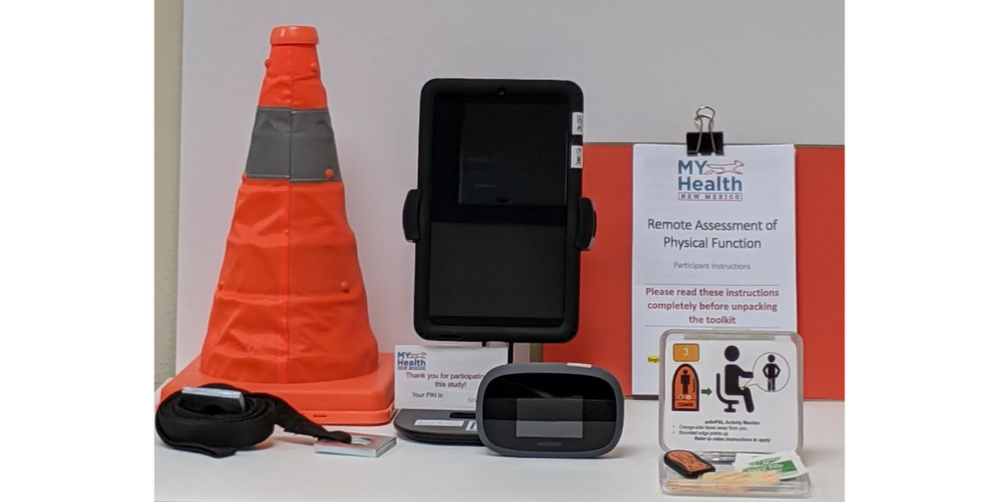
Toolkit for the remote assessment of functional mobility and strength among older cancer survivors (left to right: orange pop-up cone with black 10-foot measurement tool; tablet in tablet stand; Wi-Fi mobile hotspot; instruction booklet; activPAL activity monitor kit with alcohol wipes and adhesives).

The test kit includes an Android tablet, a Wi-Fi hotspot, a tablet stand, an activity monitor, and a measurement tool with a pop-up cone to mark 10 feet for the TUG test (Figures 2 and 3). A written instruction booklet includes information on how to set up the tablet and wireless internet hotspot, attach the activity monitor, set up their test area, accept the videoconference call, and repack the toolkit. Participants are also asked to watch a video on their tablet, which demonstrates the performance tests, safety measures, and instructions for setting up their test area. Further instructions are provided by the investigator (remote assessor) via videoconferencing before conducting the assessment.

Due to the potential for video lag or stutter during the videoconferencing call, participants wear an activity monitor during their physical performance tests. The activPAL3 is a small, light-weight research-grade monitor (2.4 × 4.3 × 0.5 cm; 10 g) worn on the thigh (PAL Technologies, Glasgow). The device includes both an inclinometer (to detect a change in position) and a triaxial accelerometer (to measure acceleration). The activPAL3 provides accurate measures of sitting (or lying), standing, and stepping [52-55]. The time-stamped data (sitting, standing, and stepping) is used to assess the measurement validity of the videoconferencing method to assess physical performance. Both written and video instructions are included to instruct study participants on how to attach the ActivPAL3 monitor. The monitor is attached to the anterior midline of the thigh (dominant leg) using a PALSticki (a double-sided hypoallergenic sticky pad).

#### Videoconferencing and Recording Software

Skype was selected as the video conferencing software due to the low-cost (free version), ease of use, and potential for familiarity among the study population. Videos are recorded and saved for quality control and to allow intra- and interrater reliability testing. During the proof-of-concept phase, it was discovered that the stored videos were cropped, reducing the field of view of the test area. Therefore, the software to record and store the videos was changed to SnagIT, a low-cost screen capture and recording software.

The remote assessor, located on campus using a reliable wireless internet connection, initiates the Skype call with the study participant. During the call, the remote assessor reviews the safety checklist with the participant and verifies the testing space is adequate and safe for conducting the two tests of functional mobility and strength. The remote assessor reviews the instructions for the tests, answers any questions, verifies activity monitor application, and tells the participant when to start/stop each test.

### Procedures: Study Phases

The study objective is achieved over a series of phases (Figure 1). We are applying a similar concept of “saturation” as is done in qualitative studies. In qualitative studies, the number of focus groups or interviews is based on the saturation point, that is, the point at which no new information is learned. For the current study, we include a range for the number of participants to be included in each study phase. At the point at which no new information is learned, ie, no further adjustments are needed to the test protocol, and we proceed to the next phase.

#### Phase 1: Proof-of-Concept

The first phase is a proof-of-concept that participants can follow the testing protocol and use the tablet PC to communicate with the investigator. The investigator tracks technology issues (use of a tablet, cellular reception, audio, and video quality) and nontechnology issues (understanding of test instructions, safety issues) during the home visits (direct observer). The investigator observes the participant unpacking the toolkit, reviewing the written and video instructions, applying the activity monitor, setting up the test course, communicating with the remote assessor, performing the gerontologic tests, and repacking the toolkit. Once the assessment is complete, the participant is debriefed by the direct observer. The debriefing opens with broad questioning that captures a participant’s general comments on how the assessment could be improved, and then specifically addresses any concerns regarding safety, the clarity of both written and verbal instructions, and comments about the toolkit (contents, packaging). The test protocol and instructions are refined based on what is learned during the proof-of-concept phase (10-12 participants).

#### Phase 2: Measurement Validity

Next, 10-20 new participants are enrolled to evaluate the criterion validity of measuring physical performance via videoconference compared to the existing gold standard, direct observation (in-person observation and scoring of the tests). We are testing whether the remote assessment via videoconferencing produces similar measurements (ie, test scores) as the traditional (direct observation) method. While one investigator is in a private office on campus with excellent Wi-Fi service conducting the videoconference assessment (remote assessor), another investigator is in the participant’s home to directly observe and evaluate the barriers and impediments related to the technology and test protocol (direct observer). The performance tests are assessed simultaneously by the two investigators to eliminate intraparticipant variation that would occur with sequential assessments. A simultaneous assessment (direct observation and remote) vs sequential assessments avoids learning effects (among high performers) and fatigability (among low performers). The remote assessor communicates with the participant during the assessment, provides instructions, and starts, stops, and times each test using a standard stopwatch. The direct observer times each test based on the remote assessor’s cues. Communication occurs between the remote assessor and the participant. The direct observer does not communicate directly with the participant, unless there is a safety issue (eg, pet walking through the test course, creating a tripping hazard), until the end of the assessment. At the end of the assessment, the participant provides feedback, which is used to improve the test instructions or protocol (as described in Phase 1). After completing assessments with 10 participants without a major change in the test protocol, we will proceed to the next phase.

#### Phase 3: Reliability

This phase involves participants repeating the test protocol, but without an investigator in their home during the assessment (ie, no direct observation). This phase tests the ability of the participant to receive the box of test instructions and materials in the mail, unpack the box, set up their test area, communicate with the remote assessor via videoconferencing, pack up the box, and return it (postage paid) to the study team. By eliminating the home visit with the direct observer, participants will have more time to review the test instructions, set up their test course, and will be restricted to communication with the remote assessor. This step involves 5-10 participants from Phases 1 and 2, who provide approval for future contact and express interest in repeating the assessment. Since these participants will have already completed the study, we anticipate the test instructions should be sufficient for the participants to safely self-administer the two physical performance tests in their own home, while communicating with the remote assessor via videoconferencing. Otherwise, further improvements/clarifications to the test instructions will be made. Once five participants have successfully and safely completed the test protocol, we will proceed to Phase 4.

#### Phase 4: Remote Assessment

This phase is the same as Phase 3, except it includes newly enrolled participants who will complete the revised test protocol and undergo de novo remote assessment of physical performance to eliminate the practice effect that is likely to occur in Phase 3. The enrollment goal for this phase is 5-10 participants. The goal of this phase is to have a finalized test protocol, toolkit, and instructions that older cancer survivors find acceptable to self-administer and safely perform the two tests of functional mobility and strength in their homes.

For safety purposes, a friend, neighbor, or relative is requested to be present during the assessment. This person can communicate via the videoconferencing session with the investigator and must have access to a telephone should medical attention be required. As needed, this person may assist the study participant with reading/understanding the instructions and setting up the test course. If this person is unable to be present during the assessment once it has been scheduled, then the research investigator conducting the direct observation covers their responsibilities (Phases 1 and 2). Otherwise, the assessment is rescheduled (Phases 3 and 4).

The anticipated number of enrolled participants ranges from 30 to 52. For each phase of the study, participants receive a $50 gift card for the completed assessment to compensate them for their time and participation.

### Subjective Measures

Sociodemographics, health-related characteristics, and medical history are used to characterize the study population. Sociodemographic data collected by the survey include age, sex, race/ethnicity, education, income range, and marital status. Health-related characteristics include smoking status, and self-reported height and weight (used to calculate body mass index (BMI; kg/m^2^). Cancer data are obtained via self-report (cancer type, year of diagnosis, and treatment received (yes/no): surgery, chemotherapy, radiation, hormone therapy). The Self-Administered Comorbidity Questionnaire [56,57] is used to assess the number of medical conditions and their impact on usual activities.

The full version of the Falls Efficacy Scale–International is completed by all enrolled participants for comparison with the shortened version used for screening. Respondent choices for concerns about falling while performing each of the sixteen activities include “not at all concerned,” “somewhat concerned,” “fairly concerned,” and “very concerned.” The scores on the full version of the questionnaire ranged from 16 to 64. Prior studies have considered a score of 24 and above as having a high concern of falling [38,58]. Fall risk assessed from the full version of the questionnaire will be used to characterize the study population.

The PROMIS-29 Profile [59] is a combination of short-forms designed to assess patient-reported outcomes across a variety of chronic diseases, including cancer [60-63]. It includes four items from seven domains (anxiety, depression, pain interference, fatigue, sleep disturbance, satisfaction with participation in social roles, and physical function) using a 5-point Likert-type scale; 1 item for pain intensity (an 11-point rating scale). Scores are normed to a general population. These instruments, developed by the NIH, have strong validity, reliability, and are responsive to change [60,62,63]. This quality of life data will also be used to characterize the health and well-being of the study population.

### Analyses

Descriptive statistics will be used to describe the sociodemographic characteristics, health-related factors, fall risk, and health-related quality of life of the study population.

We will evaluate the criterion validity of videoconference vs direct observation of the tests of functional mobility and strength. Both performance tests are timed tests (time in seconds to complete the TUG test; number of chair stands in 30 seconds). The validity of the videoconference assessment will be evaluated by estimating the limits of agreement between the two methods [64], ie, the interval containing 95% of the between-measures differences between measurements. The videoconference assessment will be considered valid if the limits of agreement are within a clinically acceptable limit [64]. This limit will be determined *a priori* based on the results from the interrater reliability evaluation using data collected during the proof-of-concept (Phase 1 of the study including the first 10 participants), thus providing a better idea of interrater differences in timing these tests under ideal conditions, ie, both investigators observing the tests from the saved video recording. The clinically acceptable limit will need to take the interrater difference into account, ie, a limit that is at or above the interrater difference under ideal conditions.

Intra- and interrater reliability testing will occur 6-8 weeks after completion of the home visit (Phases 1 and 2). The investigator who performed the videoconferencing assessment will watch the video recording and re-score the tests to determine intrarater reliability. Two different investigators will watch the video recordings and score the tests (using a stopwatch) to determine interrater reliability. All investigators will receive specific training in how to time the performance tests in the same way. We will calculate ICC to examine intrarater and interrater reliability of the videoconference assessments of physical performance. We hope to achieve ICCs that reflect good to excellent reliability (ICC >0.59) [65].

We will also evaluate the validity of videoconference vs accelerometer data collected from the physical performance tests. ActivPAL3 data will be downloaded using the activPAL software (version 8; PAL Technologies Limited). Attempts will be made to synchronize the event files to the video in order to obtain objective measures of the degree to which video assessments might accurately reflect actual motion recorded objectively by other means. The event files include the start and stop time for each sitting, standing, and stepping event, and the duration of each event. The event file (CSV file) will be processed using the activPAL Processing R package (version 1.0.2) [66,67]. Once the sit-to-stand and stand-to-sit transitions have been identified (derived from activPAL’s inclinometer data), we will use similar methods as Pickford et al to use the peak angular velocity of thigh rotation (derived from activPAL’s acceleration data) to accurately determine the start and end of the movements for both the TUG test and the 30-s CST [68].

## Results

Enrollment began in December of 2019 and is ongoing. Phase I was completed in February 2020. Proof-of-concept that participants can follow the testing protocol and use the tablet PC to communicate with the investigator was established with 10 cancer survivors, aged 70.5 (SD 6.5) years. The test protocol was refined based on what was learned in this phase, which primarily comprised enhancements to both the written and video instructions per participant feedback. Ten participants have been enrolled in the Validity Phase (Phase 2); however, only 5 participants completed the assessment before the study was paused due to COVID-19. Preparations are underway to resume the study while taking precautions to keep participants and the study team safe. The study is expected to be completed by the end of 2020.

## Discussion

Physical functioning is an important predictor of future disability, loss of independence, and premature mortality. Thus, physical functioning is frequently targeted in interventions for older cancer survivors, especially those with comorbidities or existing functional limitations. To our knowledge, low-cost, valid, reliable, and easy to use methods to remotely assess physical performance in older adults in the home environment are not readily available to the research community. Soon, wearable sensors and smartphone apps will likely meet these criteria. In the meantime, videoconferencing has a role to play in remote assessment, with the added benefits of verification of a safe environment for conducting the physical performance tests, and of successful completion of the tests, including adherence to the test instructions by study participants. The current study is evaluating the validity and reliability of using videoconferencing to remotely assess two tests of functional mobility and strength, self-administered by older cancer survivors in the home setting. While this study was designed to reduce travel burden to a clinical site for assessment with rural participants in mind, the COVID-19 pandemic underscores the need for remote assessment.

Our test protocol provides a complete toolkit and instructions to assist older cancer survivors of varying proficiency with technology, to videoconference with an investigator during the remote assessment. The simplicity of this test protocol is both a limitation and a strength. Our test protocol includes a limited number of performance tests, the TUG, and 30-s CST, which are considered basic, yet standard gerontologic performance tests. This protocol could easily be modified to include other similar tests, such as the 5-times sit to stand test or the 8-foot usual walk [12]. However, more advanced balance tests (eg, tandem stance [12], especially with eyes closed) and endurance tests (eg, two-minute step test [13,69]), would require additional safety measures. Training of the family member/caregiver on how to spot the participant during these tests would also help reduce the risk of falls during more advanced tests; however, this would require careful attention to the age and health status of the individual spotting the participant. We excluded individuals at high fall risk, primarily associated with basic activities performed at home (eg, cleaning the house and walking around in your house), rather than exclusion based on less common and more avoidable activities (eg, walking on a slippery surface or an uneven surface). Individuals at high risk for falls would likely require a more elaborate test protocol, especially for safety measures, including spotting during the tests by a trained family member/caregiver, and a physical therapist or someone with relevant clinical expertise to serve as the remote assessor.

Another limitation is the lack of standardization of the space and equipment used for testing within individual homes. Many homes are not conducive for this type of testing, especially older or smaller homes, which may have smaller rooms, doorways, and walkways, or furniture that is not easily moved. Another issue for mobility tests is having adequate space without transitions between the type of flooring (eg, tile or hardwood to carpet) or level of flooring (eg, sunken living rooms). While an individual’s normal daily activities include movements associated with many of the basic performance tests (eg, standing up from a chair, walking a short distance, turning around), the combination of movements at a faster speed (due to timed tests), especially in a crowded space, requires consideration. Videoconferencing allows for a more thorough examination of the test space and other factors affecting safety (eg, pets or children entering the test area during testing), compared to remote assessment via sensors or apps. The goal of our protocol is to strike the right balance between safety and meaningful data collection.

It is important to note that our study design and protocol were created before the COVID-19 pandemic, and thus represent a preliminary evaluation of safety and acceptability of self-administered performance tests by older adults in the home setting. Future directions include the expansion of the current protocol to include the evaluation of additional performance tests. Additionally, we anticipate that soon, low-cost, easy to use, valid and reliable sensors and apps will be available for widespread use by the research community after final evaluation and usability testing in the home environment. The sensors and apps will be able to provide greater detail and more precise measurements of the timed tests, as well as the quality of movement during the different phases of the test. Nevertheless, videoconferencing would still be of value to verify that the test area is acceptable in terms of safety and space, the test equipment has been set up properly, and the participant is wearing appropriate footwear (eg, sturdy walking shoes with nonslip soles). Additionally, videoconferencing would allow verification that the test subject is performing the tests appropriately and safely, and would allow the remote assessor to stop the test early if necessary.

This protocol was developed for use in a nontherapeutic research setting. Nevertheless, our protocol could be easily adapted to allow therapists and healthcare professionals to follow-up with their aging patients in the home setting. The current toolkit includes a tablet computer and portable wireless hotspot, which allows individuals without a portable device containing reliable, high-speed internet to participate in video conferencing. A smaller toolkit could be used for individuals who own and are comfortable using a laptop, tablet, or smartphone for videoconferencing with a health professional. Conversely, additional equipment, such as a hand dynamometer to measure grip strength, could be included in the toolkit. The cost of the contents of the toolkit may warrant insurance, should the toolkit be stolen (eg, porch pirates).

This feasibility study will provide important information on the validity, reliability, acceptability, and safety of using videoconferencing to remotely assess physical performance tests, self-administered by older cancer survivors in the home setting. If feasible and safe, this remote assessment protocol and toolkit will provide a low-cost and easy to use method to collect objective data for an important measure of physical health in older cancer survivors. The TUG and the 30-s CST are standard gerontologic tests that are responsive to change, and thus represent useful tests for interventions aiming to improve physical performance. Remote assessment eliminates travel burden for the participant and is cost-effective. Furthermore, this method will allow future interventions to expand the reach to rural, older cancer survivors, an underserved population.

## Appendix

Multimedia Appendix 1 Checklist provided to older cancer survivors prior to the remote assessment of two tests of functional mobility and strength (Timed Up and Go test, 30-second chair stand test).

## Abbreviations

30s-CST: 30-second chair stand test
ICC: intraclass correlation coefficient
STEADI: Stopping Elderly Accidents, Deaths, and Injuries
TUG: timed up and go test
