# Supplementary material for: Remote Assessment of Functional Mobility and Strength in Older Cancer Survivors: Protocol for a Validity and Reliability Study
Source: JMIR Res Protoc. 2020 Sep 1;9(9):e20834. doi: 10.2196/20834 (PMC7492978; doi:10.2196/20834)
Supplement: Multimedia Appendix 1 [file resprot_v9i9e20834_app1.docx]

Location & Materials Checklist

You will need an area of at least 12 feet by 4 feet to conduct your physical function assessment. Use the following checklist to ensure the location you choose is safe for you to conduct the tests.

- Select a chair with minimal padding without wheels (casters) to be used for both assessments (i.e. dining or kitchen chair).
- Chair should be standard height (seat height of approximately 17-inches from ground)
- Ensure adequate space to conduct the test ( a walking path of at least 12 x 4 feet)
- Testing space should be same surface throughout, such as all carpet or all hard flooring. You should not have to navigate transitions between types of flooring (i.e. hardwood to carpet/area rug) during your physical function tests
- If testing on a hard-surface, the chair back should be placed against a wall or solid table to prevent it from sliding backwards or tipping during the test
- If conducting the test on carpet or rug, make sure it is adequately affixed to floor (no bunching, sliding, or fringe)
- Clear walking path. Clothing, toys, cords should be put away
- Pets and small children should be kept away from testing area during assessment
- Make sure that your walking path and testing area are well lit
- You will need sturdy yet comfortable walking shoes with non-slip soles and solid backs (good: tennis shoes; bad: flip-flops, slides, slippers)
- Eye glasses (if normally worn)
